# Evaluation of Adiponectin and ANGPTL8 in Women With Metabolic Syndrome in the Madinah Region of Saudi Arabia

**DOI:** 10.7759/cureus.44219

**Published:** 2023-08-27

**Authors:** Walaa Mohammedsaeed, Ahmed Ahmed, Nada Alharbi, Amjaad Aljohani, Razan Alruwaithi, Reem Alharbi, Shatha Alahmadi

**Affiliations:** 1 Department of Medical Laboratory Technology, Faculty of Applied Medical Science, Taibah University, Madinah, SAU

**Keywords:** obesity, insulin resistance, mets, adiponectin, angptl8

## Abstract

Objective: *“*Metabolic syndrome” (MetS) is a set of abnormalities that may be risk factors for cardiovascular disease (CVD) and diabetes. The current study sought to (1) determine MetS prevalence and (2) examine Adiponectin and ANGPTL8 levels in connection to MetS components and CVDs and diabetes risk in females with MetS.

Methods: A total of 350, 20-35-year-old Saudi females were studied. Waist circumference (WC), body mass index (BMI), glucose, HbA1c, insulin, lipid profiles, and blood pressure (BP) were examined for MetS. ANGPTL8 and Adiponectin were also measured.

Results: The patients were classified into two groups, namely MetS and non-MetS, according to the criteria established by the National Cholesterol Education Program Adult Treatment Panel III (NCEP-ATPIII). We examined biomarker and anthropometric results between these groups. One hundred forty-four of 350 female participants (41.2%) had MetS, with a mean age of 30.5 years. Fasting blood glucose (FBG), high-density lipoprotein cholesterol (HDL-C), triglycerides (TG), ANGPTL8, adiponectin, and insulin resistance (IR) were statistically significant differences observed between the two groups. BP, BMI, WC, and Atherogenic Index of Plasma (AIP) all changed significantly (P ≤0.05). Correlation studies linked MetS components to higher ANGPTL-8 and reduced adiponectin. The levels of ANGPTL8 were shown to be influenced by the increase in FBG, TG, BP, IR, and AIP (P < 0.05). Factors such as FBG, BMI, WC, and IR have been found to have an inverse relationship with adiponectin levels.

Conclusion: 41.2% out of 350 Saudi females at Taibah University in the Madinah region had MetS, medium CVD risk, and slightly elevated BMI, TG, WC, and BP. To lower their risk of CVD and diabetes later in life, overweight young women should be evaluated for MetS. FBG and TG were substantially associated with ANGPTL8 while reducing adiponectin was associated with elevated TG and BP. Our findings may lead to ANGPTL8 and adiponectin's possible predictive function for CVD in early MetS in females.

## Introduction

Metabolic syndrome (MetS) includes a cluster of metabolic disorders characterized by elevated insulin levels, excessive body weight, hypertension, and dyslipidemia [1]. The consumption of excessive amounts of glucose has been found to elevate the likelihood of developing diabetes, cardiovascular disease (CVD), and liver and kidney diseases [2]. This condition is widely recognized as a prevalent syndrome on a global scale. MetS has been defined by various international health organizations, including the World Health Organization (WHO), the National Cholesterol Education Program-Adult Treatment Panel III (NCEP-ATPIII), the International Diabetes Federation (IDF), and the European Group for Study of Insulin Resistance [3-5]. Despite the differences in these definitions, the core complications of MetS remain largely consistent, encompassing CVD, type 2 diabetes, dyslipidemia, and hypertension. MetS is a significant public health concern in the Kingdom of Saudi Arabia (KSA), necessitating focused care and attention due to its widespread prevalence throughout the kingdom. In 2005, the prevalence of MetS in Saudi Arabia reached 39.4%, positioning Saudi Arabia among the countries with the highest global prevalence of this condition [6]. Furthermore, Saudi Arabia is recognized as one of the leading nations worldwide with high rates of diabetes and obesity, affecting over one-third of the adult population, and the incidence of these conditions continues to rise [6].

Adiponectin (ADPQ) is primarily secreted by adipocytes, with its presence in the bloodstream being predominantly cleared by the liver. The ADPQ protein complex is composed of various multimeric structures, encompassing both low molecular weight (LMW) hexamers and high molecular weight (HMW) hexamers [7,8]. According to Robinson et al., the HMW multimer is widely recognized as the primary active functional constituent of ADPQ [9]. Prior research has demonstrated that ADPQ has an impact on the maintenance of energy homeostasis through the regulation of glucose and lipid metabolism [9,10]. ADPQ has been found to have the ability to reduce insulin resistance (IR) through the activation of specific proteins, such as adenosine monophosphate-activated protein kinase (AMPK) and peroxisome proliferator-activated receptor gamma (PPAR-γ). This activation leads to an increase in cellular glucose uptake and the oxidation of fatty acids in both organs and the bloodstream [9-12]. ADPQ has been identified as a reliable biomarker for the accurate prediction and risk categorization of IR and its associated complications, as demonstrated in studies conducted by Tschritter et al., Yamamoto et al., and Hara et al. [13-15].

Angiopoietin-like proteins (ANGPTLs) consist of eight members (ANGPTL1-8) and are classified as secreted glycoproteins [16]. Numerous epidemiological studies have provided evidence of the modification of ANGPTL8 levels in metabolic disorders such as diabetes, obesity, and MetS. Previous studies have also documented the association between ANGPTL8 and other biomarkers related to these diseases. Despite the lack of consistency and the need for further clarification [17], the results of this study indicated that ANGPTL-8 may serve as a potential indicator for the presence of the condition and may have implications for its subsequent monitoring and evaluation, suggesting that the regulation of ANGPTL8 expression has the potential to serve as a novel pathway for the normalization of lipid and glucose metabolism disorders [17].

However, there is limited available data on the prevalence of MetS in the KSA, and there is a lack of research specifically focused on university populations. Only two articles have been found that examine MetS in university students, one conducted at King Saud bin-Abdulaziz University for Health Sciences using the IDF criteria [18], and another at King Abdulaziz University using the NCEP-ATPIII criteria [19]. However, no studies have been conducted on the prevalence of MetS among students and employees at Taibah University. Therefore, the objective of this study is to evaluate the prevalence of MetS in female students at Taibah University. Furthermore, considering the strong correlation between ADPQ or ANGPTL8 and the risk of MetS complications, our objective is to assess the levels of these biomarkers and establish their associations with the individual components of MetS in female subjects.

## Materials and methods

Study population

A cross-sectional study was performed at an academic institution, including individuals between the ages of 20 and 35 who were female and were either students or employees. The participants were selected using a random sample method, with the additional requirement of having no pre-existing medical issues, as indicated by the submitted information or the status of lactation or pregnancy. A cohort of 350 female participants provided informed consent to partake in the study and were entrusted with the responsibility of conducting all blood analyses. The research obtained ethical approval (SREC/AMS 2020/41/CLD) from the Institutional Review Board (IRB) of the Faculty of Applied Medical Sciences at Taibah University in Madinah, Saudi Arabia. The board in question demonstrated compliance with the regulations stipulated in the Declaration of Helsinki of 1964, as well as its subsequent amendments.

Laboratory investigations

Biochemical Parameters

Blood samples were subjected to analysis at the laboratories of Madinah Hospital following an overnight period of fasting. The parameters assessed included glucose levels, HbA1c levels, insulin levels, lipid profile, and hs-CRP levels. Subsequently, a centrifugation process was performed on a residual blood sample, utilizing a force of 3,000 times the acceleration due to gravity for a duration of 5 minutes at 37 °C. The parameter levels were determined utilizing the Cobas b 311 immunoassay analyzer in accordance with the guidelines provided by the manufacturer (Roche Diagnostics, GmbH, Germany). The reference range values employed in this study were derived from data collected from the laboratories of Madinah Hospital in the Madinah region of Saudi Arabia.

The resulting serum was then stored at a temperature of -20 °C for the purpose of analyzing the levels of ANGPTL8 (with a reference range of 0.18-3.7 ng/mL) and ADPQ (with a reference range of 5-37 µg/mL). The hormone levels were quantitatively measured using an ELISA-based chemiluminescent assay provided by CUSABIO Technology LLC, located in Houston, USA. The procedures were carried out in accordance with the instructions provided by the manufacturer.

Blood Pressure (BP)

The BPs, both systolic and diastolic, were assessed using a standardized sphygmomanometer (Baumanometer, Model 0320, W.A. Baum Co., Inc. USA). The BP measurements were taken in millimeters of mercury (mm/Hg) while the participant was in a seated position. Two measurements were obtained, with a 5-minute interval between each measurement. The average of the two measurements was then calculated. The accepted range for BP is typically defined as below 120/80 mm Hg. However, BP measurements falling within the range of 120-129 mm Hg systolic and 80 mm Hg diastolic are categorized as Elevated. Participants were classified as having stage I hypertension, also known as prehypertension, if their systolic BP (SBP) or diastolic BP (DBP) was found to be between 130-139 mm Hg or 80-89 mm Hg, respectively [20].

The assessment of IR was conducted using the homeostasis model estimation of IR (HOMA-IR) index, which is calculated by multiplying the fasting insulin level (Uµ/mL) by the fasting glucose level (mg/dL) and dividing the result by 405. The range that is deemed to be within the realm of good health is defined by values ranging from 0.5 to 1.4. Values below 1.0 indicate insulin sensitivity in patients, which is considered to be optimal. Values higher than 1.9 indicate early IR, while values above 2.9 indicate serious IR [21].

The Atherogenic Index of Plasma (AIP) was assessed by evaluating the logarithm base 10 of the ratio of triglycerides (TG) to high-density lipoprotein cholesterol (TG/HDL-C). The AIP is categorized into three risk levels for CVD: low risk, which is defined as AIP values below 0.1; medium risk, which encompasses AIP values ranging from 0.1 to 0.24; and high risk, which includes AIP values exceeding 0.24 [22].

Anthropometric Measurements

The estimation of BMI was conducted using an electronic scale manufactured by (Beurer GmbH, specifically the Type PS 07 model from China). The body mass index (BMI) has been classified into three categories: (a) underweight, which includes values less than 18.5 kg/m^2^, (b) normal, which encompasses values ranging from 18.5 to 25.0 kg/m^2^, and (c) overweight or obese, which includes values ranging from 25.0 to 29.9 kg/m^2^ and values greater than 30.0 kg/m^2^, respectively [23]. The waist circumference (WC) was measured at the level of the umbilicus. Females whose WC measures more than 88 cm were classified as individuals with abdominal obesity [23].

Definition of MetS

Several international organizations have provided various definitions for MetS, including the World Health Organization (WHO), the NCEP-ATPIII, and the IDF. The present study employs the NCEP-ATPIII criteria, which are used to determine the presence of MetS in individuals. According to these criteria, an individual is classified as having MetS if they meet three or more out of the five specified criteria. (1) Abdominal obesity can be determined by assessing waist circumference exceeding 88 cm in females. Hypertriglyceridemia is defined as a condition characterized by triglyceride levels equal to or exceeding 1.7 mmol/L. In females, a reduction in HDL-C levels below 1.3 mmol/L was observed. Hypertension is defined as having a BP reading equal to or exceeding 134/85 mm/Hg. A fasting plasma glucose level equal to or greater than 6.1 mmol/L.

Statistical analysis

GraphPad Prism 7 was used for statistics (GraphPad Software, CA, USA). Continuous data were reported as mean ± standard deviation (SD) and categorical data as numbers (%). An Independent Student t-test was used to compare research categories. Pearson's estimate test to evaluate the association between ANGPTL8, ADPQ, and the risk of MetS complications. Multiple linear regression was utilized to investigate serum ANGPTL8 and ADPQ impact on MetS components complications risk. Serum ANGPTL8 and ADPQ levels were used as the independent variable for MetS complications such as CVD along with several dependent variables (MetS components). Multiple linear regression was run twice on serum ANGPTL8 and serum ADPQ levels in MetS women to generate unstandardized coefficients (B) with 95% confidence intervals. All differences were signified at P ≤ 0.05 or P ≤ 0.001.

## Results

Three hundred and fifty students and staff members from Taibah University in the Madinah area of Saudi Arabia made up the analyzed sample. The most noticeable characteristics of the sample are shown in Table 1. There was a median age of 26.5 years old (SD = 10.5 years). Slightly increasing BP, BMI), and AIP values were combined with slightly higher fasting blood glucose (FBG), triglyceride (TG), hs-CRP levels, ANGPTL8 level, and IR. The participants females were at an early stage of high BP, were overweight, and at a moderate risk for CVD. However, HDL-C and ADPQ levels decreased below the established reference range (Table 1).

**Table 1:** Clinical and laboratory characteristics for study groups. Values are Mean ± standard deviation. Madinah Hospital labs in Saudi Arabia provided reference range values. FBG = Fasting blood glucose, HbA1c = hemoglobin A1c, HDL-C = high-density lipoprotein, and LDL-C = low-density lipoprotein, hs-CRP = high-sensitivity C-reactive protein, BMI = body mass index, WC = waist circumference, AIP = Atherogenic Index of Plasma. *BMI (25.0-29.9 kg/m^2^) overweight **120-129 mm Hg systolic and 80 mm Hg diastolic are classified as elevated ***AIP > 0.1, medium risk of CVD

Parameters	Females’ participants, n=350	Reference range
Age (years)	26.5±10.53	-
FBG (mmol/L)	5.55±0.85	3.89 - 5.50
HbA1c (%)	4.9±0.92	4.3% - 6.0%
LDL-C (mmol/L)	3.11±0.83	2.59 - 4.11
HDL-C (mmol/L)	1.02±0.37	1.04 - 1.55
Total cholesterol (mmol/L)	4.9±1.43	5.2- 6.2
Triglycerides (TG) (mmol/L)	2.3±0.52	1.7- 2.2
BMI (kg/m^2^)	26.8±9.12*	18.5 - 24.9
WC (cm)	90±11.15	<88
hs-CRP(mg/l)	15±3.32	<10
Blood pressure(systolic/diastolic)	125/80	Systolic: less than 120 mm Hg Diastolic: less than 80 mm Hg
Fasting insulin (mIU/L)	5.24±0.99	2 - 20
Insulin Resistance (IR)	1.8±0.87	0.5 - 1.4
Adiponectin (µg/mL)	5.3±1.65	5-37
ANGPTL8 (ng/L)	4.2±0.76	0.18-3.7
AIP	0.12±0.10***	<0.1

Using the NCEP-ATPIII criteria, we identified individuals who met the criteria for MetS and those who did not. We then conducted a comparative analysis of the levels of biomarkers and anthropometric measurements between these two groups. Table 2 presents the prevalence of MetS among a cohort of 350 female participants, with a mean age of 26.5 years, using the NCEP-ATPIII criteria. The table indicates that 144 individuals, accounting for 41.2% of the sample, were diagnosed with MetS with a mean age of 30.5 years. Statistically significant differences were observed in the levels of FBG, HDL-C, TG, hs-CRP, ANGPTL8, ADPQ, and IR between the two groups, with all values having a P-value less than 0.05. Additionally, significant differences were observed in the measurements of BP, BMI, WC, and AIP, with statistical significance (all P ≤ 0.05). Furthermore, with respect to the age cohorts, there was variation in the prevalence of MetS, with higher rates observed in the 26-30 and 31-35-year age groups (20% and 18.3%, respectively), while a lower rate of 2.9% was observed in the 20-25-year age group. Based on the BMI and AIP values, it was observed that the participants diagnosed with MetS exhibited a higher body weight and were classified as overweight. Additionally, they were found to have a moderate risk of developing CVD, accompanied by slightly elevated BP. Conversely, the participants without MetS displayed normal BMI values and were associated with a lower risk of CVD, as well as normal BP levels (Table 2).

**Table 2:** A comparative analysis of baseline characteristics among participants with metabolic syndrome (MetS) and those without metabolic syndrome Values are Mean ± standard deviation, and number (percentage %); P-value obtained from Independent Student t-test. *P<0.05, **P<0.001. FBG = Fasting blood glucose, HbA1c = hemoglobin A1c, HDL-C = high density lipoprotein, and LDL-C = low-density lipoprotein, hs-CRP = high-sensitivity C-reactive protein, BMI = body mass index, WC = waist circumference, AIP = Atherogenic Index of Plasma.

Parameters	Participants with MetS, n=144 (41.2%)	Participants without MetS, n=206 (58.8%)	P-value
Age (years)	30.5±10.13	22.5±9.11	0.04*
20-25	10(2.9%)	110 (31.4%)	
26-30	70(20%)	60(17.1%)	
31-35	64 (18.3%)	36(10.3%)	
FBG (mmol/L)	5.60±1.05	4.50±0.75	0.02*
HbA1c (%)	5.7±1.10	4.8±0.94	>0.05
LDL-C (mmol/L)	3.7±0.85	3.8±0.84	>0.05
HDL-C (mmol/L)	1.01±0.57	1.51±0.67	0.05*
Total cholesterol (mmol/L)	6.2±1.33	4.9±1.13	0.04*
Triglycerides (TG) (mmol/L)	2.7±0.82	1.4±0.52	0.04*
BMI (kg/m2)	28.5±7.65	25.3±9.11	0.02*
WC (cm)	95±11.13	87±10.15	0.04*
hs-CRP(mg/l)	15.3±4.32	10.2±3.32	0.001**
Blood pressure(systolic/diastolic)	130/85	115/75	0.02*
Fasting insulin (mIU/L)	5.44±0.89	5.24±0.79	>0.05
Insulin Resistance (IR)	1.8±0.87	1.2±0.37	0.05*
Adiponectin (µg/mL)	4.3±1.35	8.3±1.25	0.001**
ANGPTL8 (ng/L)	6.2±1.16	4.1±0.86	0.01*
AIP	0.12±0.11	0.07±0.01	0.03*

The correlation analyses provided substantial confirmation of the associations between increased ANGPTL8 levels and decreased ADPQ levels with components of MetS (Table 3). The study found a direct association between ANGPTL8 and FBG, TG, BP, IR, and AIP (all with a P-value ≤ 0.05). Conversely, ADPQ showed an inverse correlation with FBG, BMI, WC, and IR (Table 3). No significant correlations were observed between the levels of ANGPTL8 or ADPQ and HDL-C level (P > 0.05).

**Table 3:** The correlations between the level of ANGPTL8, ADPQ and MetS Components in MetS patients P-values were obtained from Pearson's correlation; Starred values point to a significant level

MetS Components	ANGPTL8		Adiponectin (ADPQ)	
	r	P	r	P
FBG (mmol/L)	0.681	0.03*	-0.521	0.05*
HDL-C (mmol/L)	0.157	>0.05	0.132	>0.05
Triglycerides (TG) (mmol/L)	0.752	0.02*	0.212	>0.05
BMI (kg/m^2^)	0.231	>0.05	-0.531	0.04*
WC (cm)	0.220	>0.05	-0.612	0.05*
Blood pressure(systolic/diastolic)	0.521	0.04*	0.321	>0.05
Insulin Resistance (IR)	0.567	0.03*	-0.581	0.04*
AIP	0.642	0.02*	0.111	>0.05

In the current investigation, ANGPTL8 and ADPQ were determined to be independent variables associated with an elevated likelihood of CVD, as evidenced by their influence on markers of MetS. Conversely, markers of MetS, including FBG, TG, BMI, and BP, were identified as dependent variables. A negative coefficient indicates that there exists an inverse relationship between the independent variable and the dependent variable, such that an increase in the independent variable corresponds to a decrease in the dependent variable. Conversely, a positive coefficient indicates a direct relationship between the independent variable and the dependent variable, where an increase in the independent variable corresponds to an increase in the dependent variable. Therefore, the linear regression analysis (Table 4) was used to examine the association between circulating levels of ANGPTL8 and ADPQ with components of MetS. The initial model indicated a significant positive association between ANGPTL8 and FBG, TG, IR, and AIP in patients with MetS (B = 6.8, 6.9, 5.8, 5.7, respectively, P ≤ 0.05). Nevertheless, a significant negative association was observed between ADPQ and the components of MetS, including TG, BP, IR, and AIP (all with a P-value ≤ 0.05, Table 4).

**Table 4:** Linear regression showing the association between ANGPTL8, ADPQ, and MetS components in MetS patients Linear regression was conducted to evaluate the association between ANGPTL8, ADPQ, and MetS Components. Unstandardized coefficients (B) and 95% confidence intervals (CIs) were statistically significant at P ≤ 0.05* or ≤ 0.001**.

MetS Components	ANGPTL8			Adiponectin (ADPQ)		
	B	95% CI	P-value	B	95% CI	P-value
FBG (mmol/L)	6.8	1.872 - 7.134	0.01*	-2.5	1.112 - 2.104	>0.05
HDL-C (mmol/L)	2.4	1.918 - 2.421	>0.05	2.1	1.898 - 2.420	>0.05
Triglycerides (TG) (mmol/L)	6.9	1.763 -8.191	0.03*	-5.8	1.273 - 6.191	0.04*
BMI (kg/m^2^)	2.8	1.115 - 3.504	>0.05	-2.6	1.165 - 2.904	>0.05
Blood pressure(systolic/diastolic)	2.7	1.216 - 3.778	>0.05	-5.3	1.216 – 6.088	0.02*
Insulin Resistance (IR)	5.8	1.703 - 7.726	0.01*	-7.7	1.781 - 8.016	0.04*
AIP	5.7	1.714 - 6.648	0.04*	-4.1	1.104 - 5.158	0.05*

## Discussion

The present investigation aimed to assess the prevalence of MetS among a sample of university students and employees aged 20 to 35 years at Taibah University. The prevalence of MetS, as determined by the NCEP-ATPIII criteria, was found to be 41.2%. This figure is notably higher than the previously reported prevalence of 27.1% in female students at Taibah University who were part of a study investigating the association between migraine and MetS [24]. The frequency observed in the present report was found to be higher compared to the frequencies reported in two previous studies conducted among university students [25,26]. The first study, conducted in Riyadh, adhered to the IDF criteria and reported a frequency of approximately 7.8%. This frequency was also higher than the frequency observed in the same study among obese students, which was reported to be 26.4% [25]. The second study was conducted in Jeddah, focusing on female students. The prevalence rate was found to be 17.7%, as determined by the NCEP-ATPIII guidelines [26]. Furthermore, the present study observed a higher prevalence of study frequency compared to a previous study conducted among young adults (aged 18-30 years) in Saudi Arabia, which reported a study frequency of 12% based on the criteria set by the IDF [27]. In the present study, the observed frequency was found to be higher among the regional population compared to female Emirate college students, which accounted for 6.8% of the sample [28], and also higher than the prevalence among obese female students, which was recorded at 34.5% [28]. Furthermore, the present frequency of the aforementioned condition exhibited a higher prevalence compared to a previous study conducted on obese Palestinian university students, where the rates were reported as 28.6% and 24% according to the IDF and NCEP-ATPIII criteria, respectively [29]. Moreover, the observed frequency was also found to be higher than the prevalence reported among students in Sudan, which stood at 7.8% [30]. Globally, our frequency exhibited a higher prevalence compared to numerous other frequencies. A systematic review conducted in the United States and South America revealed that the prevalence of MetS ranged from 0% to 19.2%, as determined by the NCEP-ATPIII-20 criteria [31]. This prevalence surpassed the frequencies reported in previous studies conducted among university students in various countries, including Kenya, the United States, India, Brazil, and Korea [32-37]. There exists a theoretical framework that posits a higher prevalence of MetS in adults compared to individuals younger than 29 years old. Our study corroborates this assertion, as we found that the average prevalence of MetS in adults residing in Saudi Arabia exceeded 28% [38]. A high prevalence of MetS is associated with significant risk factors for the development of CVD and diabetes. As a result, there is a need for effective interventions to prevent the occurrence of these conditions and subsequently reduce the associated morbidity and mortality. The high prevalence of MetS parameters in the KSA has been attributed to a primary factor, namely, an unfavorable lifestyle characterized by the consumption of unhealthy foods and a lack of physical activity. This is particularly evident in urban communities, where inadequate infrastructure, such as limited sidewalks and parks, coupled with the hot climate, further restricts opportunities for engaging in physical activity [39].

The present study aims to provide a comprehensive analysis of findings that have not been adequately explored in the existing literature. The identified potential association relates to the correlation between ANGPTL8, ADPQ, and the diverse components of MetS. The aforementioned association exhibits a notable positive correlation between ANGPTL8 and various markers, including FBG, TG, IR, and AIP. On the other hand, there exists an inverse relationship between these indicators and ADPQ.

The current study aims firstly to examine the correlation between ANGPTL8 and the various components of MetS. We determined positive associations between increased levels of ANGPTL-8 and the different components of MetS, such as FBG and TG, in the study participants. These findings align with the results reported by Lee et al. [40], who similarly concluded that individuals with elevated ANGPTL8 levels exhibited a risk of developing MetS and diabetes that was more than three times higher than those with lower levels. The study conducted by Farha et al. [41] also provided evidence indicating that individuals in the highest tertile of ANGPTL8 exhibited a significantly increased likelihood of developing type 2 diabetes mellitus (T2DM). Additionally, Leiherer et al. [42] demonstrated through multivariate analysis that ANGPTL8 emerged as an independent predictor of the risk of developing diabetes mellitus (DM). This finding has enhanced the notion that ANGPTL8 could serve as a potential predictor for the development of DM resulting from IR induced by elevated levels of ANGPTL8. However, additional research is necessary to provide further clarification on this issue. Moreover, it is widely recognized that ANGPTL8 plays a significant role in lipid metabolism [43], particularly in relation to TG, which are considered a crucial component in the development of MetS. The evidence strongly supports the existence of a notable positive correlation between ANGPTL8 and circulating TG levels, as indicated by previous research [43]. The reason for this phenomenon is attributed to the direct regulatory impact of ANGPTL8 on LPL activity, leading to a decrease in the clearance of circulating TG. Furthermore, various studies have proposed a correlation between ANGPTL8 and total cholesterol (TC), low-density lipoprotein (LDL), and high-density lipoprotein (HDL). These findings suggest that there exists an intrinsic connection between ANGPTL8 and lipid metabolism, as well as diseases characterized by lipid metabolism dysfunction [44,45]. Numerous studies have demonstrated that ANGPTL8 holds potential as a biomarker for metabolic disorders and associated diseases [45]. Moreover, elevating ANGPTL8 levels appears to have a beneficial effect on MetS. Additionally, it has been observed that the baseline level of ANGPTL8 is positively associated with all-cause mortality in individuals with T2DM including CVD [46]. The existing research indicates that ANGPTL8 may have detrimental effects, specifically, increased levels of ANGPTL8 at baseline are correlated with an increased likelihood of developing MetS. Simultaneously, the observed inhibition of ANGPTL8 has demonstrated beneficial effects, as evidenced by a reduction in the levels of circulating TG [47]. Nevertheless, the precise expression pattern of ANGPTL8 in certain pathological conditions, including obesity, diabetes, and MetS, has yet to be fully elucidated. The association between ANGPTL8 and biomarkers in these diseases is also indeterminate. Implementing stricter control over nutritional status and employing advanced techniques for measuring ANGPTL8 could potentially contribute to the resolution of the issue at hand.

Another key objective of the present study is to investigate the association between adiponectin (ADPQ) levels and the different components of MetS. A noteworthy inverse correlation was found between ADPQ and the various components of MetS, namely TG, BMI, BP, and IR. Prior research has indicated that there is a notable and gradual decline in adiponectin levels as IR and obesity levels increase [48]. This suggests that ADPQ could be a valuable tool for screening individuals for MetS [48]. Obesity plays a significant role in the occurrence of IR and is recognized as a prominent risk factor for the onset of T2DM and CVD among individuals with MetS [48]. Previous studies have demonstrated that hypoadiponectinemia occurs prior to a decline in insulin sensitivity [49] and can also serve as a predictor for the transition from normoglycemia to prediabetes [50]. The aforementioned studies and our research findings emphasize the potential utility of ADPQ as a screening modality for identifying individuals with MetS and its associated complications. The findings of the additional research emphasize that a decrease in ADPQ levels is a significant indicator of the likelihood of developing T2DM in the future [51]. There is an association between ADPQ levels and the occurrence of diabetes as well as glycemic control. Consequently, these levels may serve as valuable supplementary indicators for the screening of IR and MetS. Furthermore, it is worth noting that the dysregulated production of adipokines may serve as a potential mechanism underlying the detrimental effects of obesity. Specifically, there is an excessive production of leptin that promotes IR and inflammation, while the levels of ADPQ, which possesses insulin-sensitizing, anti-atherogenic, and anti-inflammatory properties, are diminished [52,53]. The observed decrease in ADPQ levels, which is proportional to the severity of IR and obesity, suggests that ADPQ may serve as a valuable screening tool for MetS [53]. Hypoadiponectinemia and dyslipidemia are frequently observed in individuals with obesity and the MetS that is associated with obesity. Furthermore, research has indicated that there exists an inverse relationship between plasma ADPQ levels and triglyceride concentrations, as well as a positive association with HDL cholesterol levels [54,55]. These findings suggest that ADPQ may play a role in the regulation of lipid metabolism. Consistent with our research, the majority of prior studies have indicated a negative correlation between circulating ADPQ and serum triglyceride levels [54,55]. The main cause of the increase in atherogenicity in people with hypertriglyceridemia is the rise in TG-rich lipoproteins, and this relationship has been found to be directly linked [54,55]. Hence, the increase in TG levels resulting from the decrease in adiponectin may potentially diminish the anti-atherogenic properties of ADPQ, as indicated in a previous investigation [55]. It was determined that there is an inverse association between the reduction of ADPQ levels and the elevation of BP in females diagnosed with MetS. This finding suggests that ADPQ may play a significant role in the development of hypertension. It has been previously established that ADPQ has the ability to reduce BP through both central and peripheral mechanisms [55]. This has been supported by experimental studies conducted on both animals and humans [55]. The impact of ADPQ on cardiovascular well-being has prompted extensive investigation into its potential as both a marker for cardiovascular risk and a target for therapeutic interventions. When considering the collective findings, the notable correlations between adiponectin levels and MetS indicators such as IR, TG, and BP indicate that ADPQ has the potential to serve as a biomarker for evaluating the presence of MetS and for screening for T2DM or CVD.

## Conclusions

The study conducted at Taibah University in Saudi Arabia revealed an increased prevalence of MetS among female participants. The primary factors contributing to this prevalence were elevated BMI, TG, WC, and BP. Additionally, the observed risk of CVD was classified as moderate. Given the unfavorable lifestyle practices prevalent in Saudi Arabia and the rising prevalence of diabetes and obesity, it is crucial for young adults to undergo screening for MetS parameters. Consequently, raising awareness about the implications of this syndrome becomes imperative, as it may pave the way for targeted and effective interventions aimed at reducing the risk of CVD and diabetes in the future. Furthermore, there exists a significant correlation between the elevated levels of ANGPTL8 and the various constituents of MetS, including FBG and TG. Conversely, a decrease in adiponectin level is observed, which is inversely associated with TG and BP. The results of our study could potentially pave the way for future scientific investigations into the potential predictive significance of ANGPTL8 and ADPQ in the early detection of MetS in female individuals. However, considering the fact that there are multiple inquiries that need to be addressed: Is the elevated level of ANGPTL8 and the reduced level of ADPQ in association with MetS considered a causal factor or an effect of MetS and its associated complications, such as diabetes and CVD? Hence, it is imperative to conduct extensive prognostic studies on both genders in order to elucidate the underlying mechanism of this correlation. Our research was not without limitations. The authors were unable to reach a definitive conclusion regarding the potential predictive role of ANGPTL8 and ADPQ in relation to the risks and complications associated with MetS due to the unavailability of patient follow-up. This study can be regarded as an initial investigation with the objective of comprehending the determinants contributing to elevated levels of circulating ANGPTL8 and reduced ADPQ in patients diagnosed with MetS. However, further validation of the results is necessary through subsequent studies conducted on a larger scale. These studies should employ appropriate methodologies to investigate the underlying mechanisms that connect MetS components with the levels of circulating ANGPTL8 and ADPQ.
